# Personalised *in silico* biomechanical modelling towards the optimisation of high dose-rate brachytherapy planning and treatment against prostate cancer

**DOI:** 10.3389/fphys.2024.1491144

**Published:** 2024-10-24

**Authors:** Myrianthi Hadjicharalambous, Yiannis Roussakis, George Bourantas, Eleftherios Ioannou, Karol Miller, Paul Doolan, Iosif Strouthos, Constantinos Zamboglou, Vasileios Vavourakis

**Affiliations:** ^1^ Department of Mechanical and Manufacturing Engineering, University of Cyprus, Nicosia, Cyprus; ^2^ Department of Medical Physics, German Oncology Centre, Limassol, Cyprus; ^3^ Department of Agriculture, University of Patras, Messolonghi, Greece; ^4^ Intelligent Systems for Medicine Laboratory, University of Western Australia, Perth, WA, Australia; ^5^ Department of Radiation Oncology, German Oncology Center, Limassol, Cyprus; ^6^ Department of Medical Physics and Biomedical Engineering, University College London, London, United Kingdom

**Keywords:** *in silico* modelling, meshless, simulation, brachytherapy, radiotherapy, drug delivery, preoperative planning

## Abstract

High dose-rate brachytherapy presents a promising therapeutic avenue for prostate cancer management, involving the temporary implantation of catheters which deliver radioactive sources to the cancerous site. However, as catheters puncture and penetrate the prostate, tissue deformation is evident which may affect the accuracy and efficiency of the treatment. In this work, a data-driven *in silico* modelling procedure is proposed to simulate brachytherapy while accounting for prostate biomechanics. Comprehensive magnetic resonance and transrectal ultrasound images acquired prior, during and post brachytherapy are employed for model personalisation, while the therapeutic procedure is simulated via sequential insertion of multiple catheters in the prostate gland. The medical imaging data are also employed for model evaluation, thus, demonstrating the potential of the proposed *in silico* procedure to be utilised pre- and intra-operatively in the clinical setting.

## 1 Introduction

Prostate cancer (PCa) is the second most common cancer type in men worldwide, presenting with more than one million new cases each year (Rawla, 2019). Owing to early detection and improved treatments, mortality rates have been steadily declining in western countries. Nevertheless, a trend for a worldwide increase in PCa incidence has been reported (Siegel et al., 2023), necessitating improved diagnosis and more efficient treatment strategies. Currently, common treatment options range from watchful waiting and active surveillance to radical prostatectomy, while hormone, chemo and immunotherapy are alternatives that can be used either as monotherapies, or in combination with surgery and radiotherapy (Parker et al., 2020). High-dose rate brachytherapy (HDR-BRT) is a form of internal radiation therapy for PCa, in which a radioactive source delivers high doses of radiation throughout the prostate for short periods of time (e.g., in the order of seconds to minutes). HDR-BRT has shown high potential as a therapeutic option as it can achieve similar efficiency to radical prostatectomy (Strouthos et al., 2018), is associated with excellent long-term clinical outcomes (Zamboglou et al., 2013), and requires a low number of visits to the clinic, thus, resulting into minimal disruption to the patient quality of life.

A radioactive source (typically Iridium-192) is temporarily administered to the tumour site through hollow metallic needle-like tubes, also referred to as catheters (Strouthos et al., 2022), which are inserted into the patient’s prostate. Catheter placement follows a pre-operative plan relying on detailed images of the patient’s anatomy of the prostate and surrounding organs, whereby catheter positioning is selected such that the distribution of radiation dose is maximal at the lesions and uniform throughout the prostate organ, while being minimal to surrounding tissue to reduce the effects of irradiation (further details can be found in the Supplementary Material). An important challenge in HDR-BRT is posed by the substantial deformation of the prostate, the bladder and the surrounding tissue, particularly during the needle insertion stage of the procedure. Due to the compliance of the prostate and surrounding organs, the puncturing medical instruments in brachytherapy cause tissue to displace and deform. This, in turn, has been clinically observed to introduce discrepancies between the pre-operatively planned positioning of the catheters’ tip and their actual positions. With catheter-needle insertion being a highly operator-dependent procedure, target errors up to 6 mm have been reported, which is a non-negligible error considering the scale of the problem (Xu et al., 2010). Accordingly, manual adjustments in needle positioning are often required, which are prone to errors and cause additional discomfort to patients.

Computational modelling, also referred to as *in silico* modelling, has been applied to simulate soft biological tissue biomechanics, the mechanical interaction of tissue with foreign components (e.g., catheters, stents, etc.) and predict the deformations and stresses as a result of such mechanical interactions (Carniel et al., 2020; Killeen et al., 2023). Accordingly, *in silico* modelling holds great promise to be used for pre-operative planning, or as a computer-supported intra-operative platform. Focusing on brachytherapy, *in silico* modelling can be used to ameliorate the operational errors of catheter targeting and optimise their positioning in the prostate. This can be accomplished by simulating catheter placement while accounting for the considerable deformations induced on the prostate and surrounding tissues during the operation. Models can elucidate the needle/tissue biomechanical interactions and could, therefore, be particularly helpful as a pre-operative planning tool (e.g., to select correct initial needle positioning), or as a training tool in which operators learn to compensate for the effect of needle-induced deformation.

Mathematical and computational modelling for PCa management (disease diagnosis and prognosis, PCa prediction and patient response to treatment) has seen considerable progress over the past decade. Developments span from numerical methods and procedures for prostate medical image computing and 3D model generation (Ghasab et al., 2017) to deep neural networks for the diagnosis of PCa using multi-parametric MRI data (Yi et al., 2022) and image computing algorithms based on the VERDICT model to quantify neoplasia aggressiveness by examining diffusion MRIs (Johnston et al., 2019). Another family of computer models is dedicated to forecasting of untreated prostate cancer growth at the tissue/organ scale biomechanics [e.g., the image-based biomechanical models of Lorenzo et al. (2019) and Lorenzo et al. (2022)] or at the cell-scale of the prostate tumour/host microenvironment [e.g., the agent-based biology model of van Genderen et al. (2024)], respectively. Mathematical models have also been combined with genetic algorithms to solve the optimisation problem in planning the needles position and the dose coverage on the prostate and its surroundings, while reducing the total number of needles used in the treatment (Ferrari et al., 2014). Probabilistic and machine learning models have additionally been employed towards optimising the dosage parameters in high- and low-dose rate internalised radiation therapy of PCa (Rajković et al., 2020; Chatzipapas et al., 2021). Finally, in the context of post-implant dosimetry in prostate brachytherapy, several machine learning models have been reported for seed localisation in PCa brachytherapy (e.g., Yuan et al., 2019; Younes et al., 2021). The recent review articles of Phan et al. (2020) and Morén et al. (2021) give a concise overview of the state-of-the-art mathematical models of cellular kinetics, PCa disease progression modelling and immunology, as well as optimisation models for HDR-BRT dose planning.

Due to this evident potential of HDR-BRT, significant research effort has been devoted to models of needle insertion into soft tissues, focusing on the forces developed on needles and tissue as well as on the induced soft organ deformations (Okamura et al., 2004). A common approach has been the description of forces acting on the needle tip and shaft through phenomenological models (Abolhassani et al., 2007; Oldfield et al., 2013). Tissue–needle interaction has also been simulated through prescribed constraints on tissue/needle interfaces, using Lagrange multipliers for constraint enforcement (Bui et al., 2019). Force modelling is typically motivated by experimental studies measuring needle forces in phantoms or in animal experiments (Urrea et al., 2016; Chinzei and Miller, 2001). Needle insertion has also been simulated using finite element or point-cloud based numerical methods via kinematic approaches, where the needle path is prescribed by imposing appropriate boundary conditions (Wittek et al., 2008; 2020).

Existing modelling works on needle insertion have achieved some progress in the field, e.g., haptic simulators of needle insertion for pre-surgery training (Goksel et al., 2011). Nevertheless, clinical translation of *in silico* models for procedures involving percutaneous needle insertion has been limited, largely due to oversimplifying model assumptions and to the lack of suitable data for model personalisation and validation. For instance, earlier studies typically employ the linear elasticity theory (DiMaio and Salcudean, 2003); however, prostate tissue is highly nonlinear (Ma et al., 2012), while surgical needle insertion might induce strains up to 80% (Wittek et al., 2008). Additional errors might be introduced by the employment of the finite element method–the method of choice for the solution of boundary-value problems in biomechanics–which is known to be suboptimal when very large deformations are involved due to element distortion. Importantly, the majority of published *in silico* models focus towards simulating the implantation of a single needle, whereas in HDR-BRT, as with several other medical procedures, the implantation of multiple needles/catheters (commonly 15–20 in total) is encountered in the clinical routine–a substantially more complex biomechanical problemto model and simulate. At the same time, clinical translation would be facilitated by accurate personalised predictions of needle placement. Including patient-specific geometries and data-driven boundary conditions can be instrumental for capturing the physiological deformation due to catheter-needle implantation (Misra et al., 2009), nevertheless, very few studies have focused on model personalisation. Importantly, potential clinical translation of *in silico* models requires strong evidence on their accuracy. However, validation data have scarcely been included in relevant modelling studies, with the methodology commonly assessed through phantom experiments or images depicting the insertion of a single needle.

**FIGURE 1:**
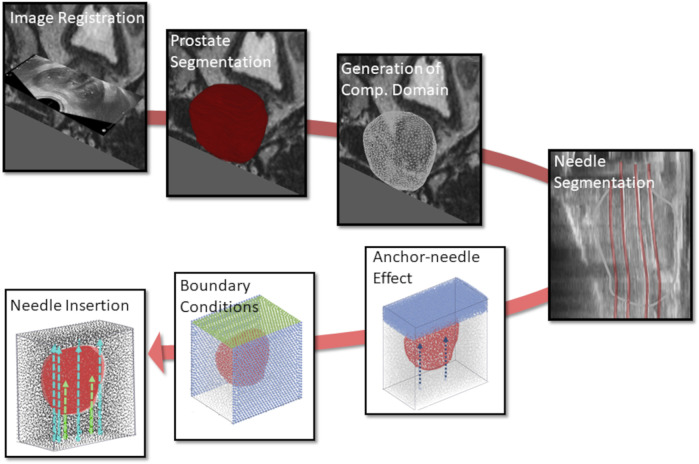
Workflow of the *in silico* simulations for patient-specific HDR-BRT modelling, involving image processing and model development tasks. Initially, image registration was performed to allow for consistent use of all available images, and the computational domain of the prostate 3D model was generated. Subsequently, (anchor and catheter) needle placement locations were identified through segmentation, which formed the boundary/loading conditions in the model. HDR-BRT simulations were initiated with the placement of the anchor needles and continued with the sequential insertion of all catheter-needles into the prostate.

In view of the evident technological gap, this work proposes a novel data-driven *in silico* modelling framework for personalised HDR-BRT simulationsof (anchor and catheter) needle insertion and prostate biomechanical modelling–the workflow is schematically laid out in Figure 1. The framework encompasses tissue deformations due to catheter insertion and is capable of providing accurate predictions of the deformed organ, on a patient-specific basis. A new kinematic approach is employed to simulate catheter-needle insertion and placement, while enhanced accuracy is achieved by accounting for the nonlinear biomechanical behaviour of the prostate gland through appropriate soft-tissue constitutive laws. Importantly, focus is placed on simulating the entire HDR-BRT procedure, involving the implantation of anchor needles that stabilise the prostate position, followed by the insertion of multiple catheters that are purposed to deliver the radioactive sources to the tumour site. Accordingly, this contribution attempts to simulate a very challenging, from a biomechanics point of view, therapeutic procedure for PCa management that has received very little attention thus far. Notably, the developed *in silico* modelling framework is informed by comprehensive state-of-the-art medical images acquired prior to HDR-BRT which provide patient-specific organ geometries and personalised boundary conditions, while images acquired during and after HDR-BRT offer invaluable information for model evaluation.

## 2 Materials and methods

### 2.1 Medical imaging data acquisition and processing

**FIGURE 2:**
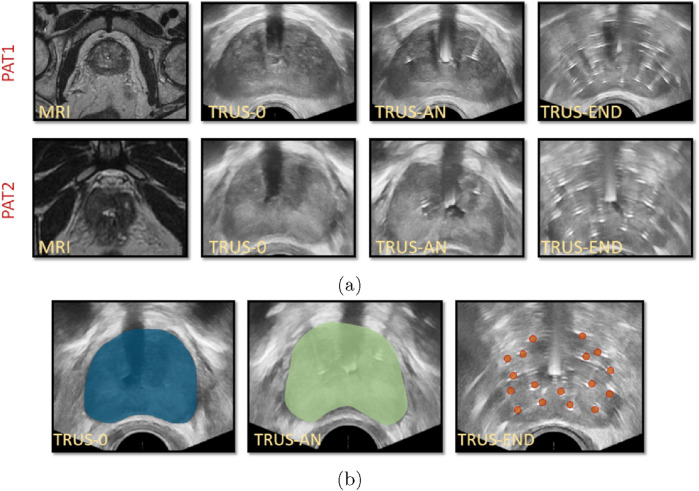
**(A)** Medical images for PAT1 and PAT2 including MRIs prior to catheter insertion, TRUS-0 (prior to catheter insertion), TRUS-AN (following the insertion of two anchor needles) and TRUS-END (ifollowing the insertion of all catheters). **(B)** Segmentation of TRUS-0 image, guided by the prostate MRI segmentation (*left*), segmentation of TRUS-AN (*middle*) and identification of catheters’ placement in TRUS-END, marked with red dots (*right*) for PAT2.

Medical images were acquired during HDR-BRT for two PCa patients (PAT1 and PAT2), at the German Oncolocy Center (GOC), with details provided in the Supplementary Material. Briefly, medical images used in this study (Figure 2A) included (*i*) an MRI scan acquired prior to brachytherapy, (*ii*) a TRUS scan acquired before initiating transperineal anchor needle and catheter-needle insertion (TRUS-0), (*iii*) a TRUS scan acquired after the two anchor needles were positioned (TRUS-AN) and, (*iv*) a TRUS scan acquired at the end of the procedure with all catheters inserted (TRUS-END).

**FIGURE 3:**
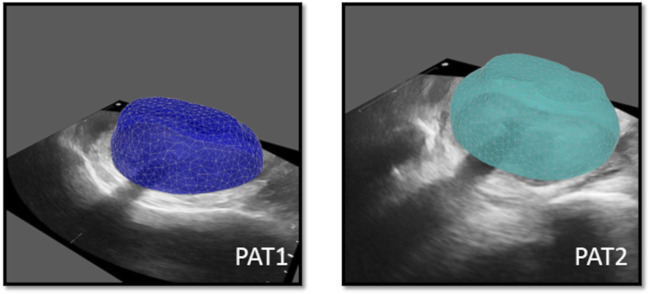
Personalised computational prostate grids for PAT1 (*left*) and PAT2 (*right*) respectively, superimposed on the respective TRUS-0 images.

Essential image processing was performed to enable model personalisation, including the spatial registration of all available images, the segmentation of the prostate anatomies and catheters (Figure 2B) and the construction of personalised grids, relying on the segmented geometries. Personalised grids (Figure 3) formed the computational domain for the HDR-BRT simulations, while segmentations of TRUS-END were essential for model evaluation. Details on the image processing followed are provided in the Supplementary Material.

### 2.2 Modelling prostate biomechanics and catheter/needle insertion

The mechanical deformation of the prostate tissue due to catheter insertion was formulated within the element-free Galerkin framework (Belytschko et al., 1994), which has proven versatile for simulations involving large strains and moving boundaries (Horton et al., 2010). The mesh-free numerical method employed builds on the meshless total Lagrangian explicit dynamics (MTLED) method of Joldes et al. (2019). Briefly, in this work the MTLED accounts for the nonlinear stress-strain behaviour of the prostate tissue (a near-incompressible Neo-hookean constitutive model was employed (Ogden, 1997)), and allows for enhanced computational efficiency and numerical convergence by combining total Lagrangian dynamics with explicit time integration, using interpolating shape functions in the framework of the modified moving least squares method of Bourantas et al. (2021). The external boundary of the simulated three-dimensional domain was constrained through Dirichlet boundary conditions while a traction-free surface was assumed on the side where the anchor needles and the catheter-needles were inserted. Detailed description of the boundary conditions and the material behaviour of the *in silico* model is provided in the Supplementary Material.

Importantly, a kinematic approach was utilised to model the insertion of a single catheter, building on the approach of Wittek et al. (2020). Briefly, a region adjacent to the catheter insertion path was assumed to move following the catheter tip, where material points in the tissue were displaced depending on their distance from the tip–further information about the formulation is provided in the Supplementary Material.

### 2.3 HDR-BRT simulation procedure

HDR-BRT began by simulating the insertion of the two anchor needles (see TRUS-AN images in Figure 2A) – the description of a typical brachytherapy clinical procedure is provided in the Supplementary Material. Clinically, the purpose of the anchor needles is to stabilise the prostate during the insertion of the catheters carrying the radioactive doses. This restriction in motion should maximise the adherence to the pre-operative plan of catheter placement and, thus, increase the treatment accuracy. In the proposed model, the effect of the anchor needles was simulated by making the region behind the anchor needles stiffer. Following the insertion of both anchor needles, the surrounding (non-prostate) tissue region behind the distal end of the needle tip was set to a higher stiffer value ( ∼30 times stiffer). The stiffer region was created to prevent substantial displacement in the direction of catheter insertion–mimicking the purpose of anchor needles.

Next step in the HDR-BRT procedure was the sequential insertion of catheter-needles–these are placed in the prostate tissue to deliver the radioactive dose to the tumour. Catheter-needles are thicker than the anchor needles so that they can carry the radioactive material, and travel to a higher depth in the prostate. Mimicking the HDR-BRT procedure, catheter-needles were inserted sequentially within the proposed modelling framework. Although the exact order of catheter insertion was not available, the order selected followed the standard clinical practice. The insertion location for each catheter was determined from manual segmentations of medical data, as described in the Supplementary Material.

Another feature included in the model was the effect of catheters already placed in the prostate geometry. Once a catheter is inserted into the prostate, a small region adjacent to it is substantially deformed and it is reasonable to assume that this region would not be considerably affected by the insertion of subsequent catheters. Accordingly, to model the effect of already positioned catheters, regions adjacent to previously-inserted catheters were set to a higher stiffness value ( ∼30 times stiffer).

## 3 Results

### 3.1 Patient-specific HDR-BRT simulations

Medical images were collected from two male patients (PAT1 and PAT2), randomly selected from a cohort with intermediate PCa risk who received HDR-BRT at GOC. Details of the HDR-BRT procedure for the two patients were recorded, including the number of anchor-needles ( × 2) and catheter-needles ( × 17) inserted. The diameter of the anchor-needles was 1 *mm*, while the diameter of the catheter-needles was 1.5 *mm*.

**FIGURE 4:**
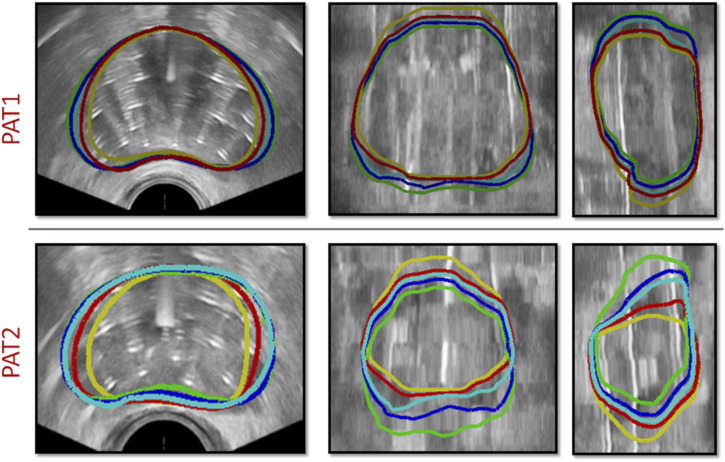
Sequence of simulations of catheter-needle insertion throughout HDR-BRT for PAT1 (*top row*) and PAT2 (*bottom row*). Yellow solid curves outline the undeformed prostate geometry, i.e., from TRUS-0, while the deformed prostate geometry once the two anchor-needles have been inserted is shown in red. Cyan, blue and green solid curves outline the simulated prostate geometry following the insertion of the 5th, 10th and 17th catheter-needle respectively.

The location of needle insertion, along with the depth each needle had travelled through the prostate tissue was determined after delineating the catheters using the TRUS-AN and TRUS-END images. Patient-specific catheter positions and prostate geometries were incorporated into the personalised HDR-BRT simulations for the two patients. For each patient, as explained in Section 2.3, HDR-BRT was simulated in a sequential manner, whereby the first and then second anchor-needles was inserted, followed by the sequential insertion of 17 catheter-needles. Figure 4 captures a sequence of simulated deformation outcomes for the prostate tissue as the needle insertion process in HDR-BRT progressed.

**FIGURE 5:**
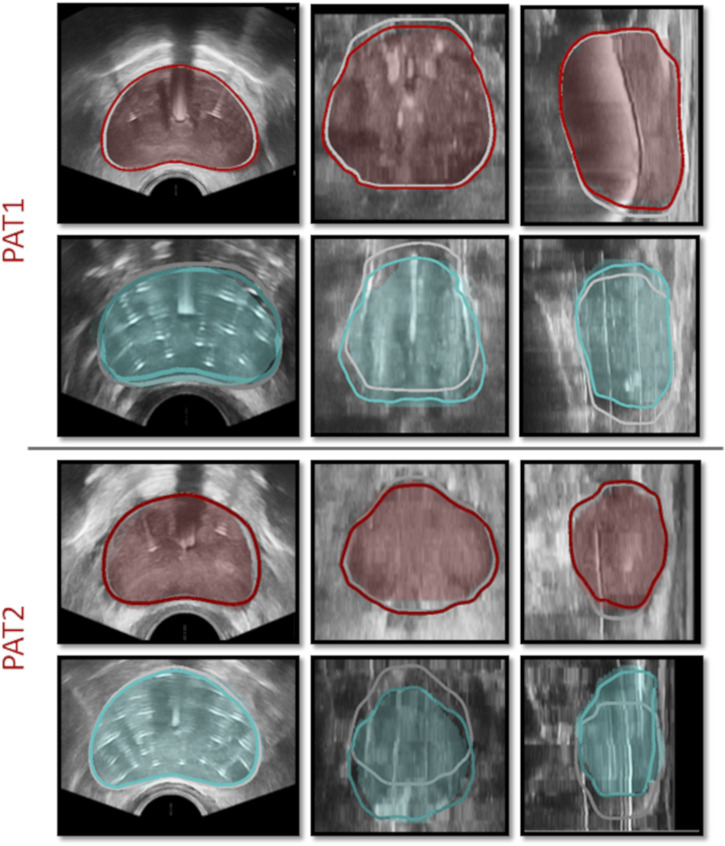
HDR-BRT simulations once the anchor needles have been inserted (*1st and 3rd row*) and once all catheter-needles have been inserted (*2nd and 4th row*), for PAT1 and PAT2. The contours mark the simulated prostate, while the shaded regions signify the segmented prostate domain from TRUS-AN and TRUS-END. Grey solid curves outline the prostate in the initial undeformed (TRUS-0) configuration.

TRUS-END images acquired after all needles were inserted were used to assess the *in silico* model predictions. Qualitative comparisons were performed by comparing the simulated deformed prostate geometry against segmentations of the prostate geometry from TRUS-END–these segmentations were produced manually by adjusting the contours of the TRUS-0 images. Figure 5 illustrates the simulated deformation for both patients, once the two anchor needles have been inserted and once all catheter-needles have been inserted and the catheters installation process has been finalised.

Quantitative comparisons were also performed by evaluating the Dice similarity coefficient, D , for each case. The agreement between the *in silico* predictions and the ultrasound images was assessed by evaluating D on a common cubic image domain, surrounding the simulated deformed prostate geometry and the segmented masks from TRUS-END (Table 1). The cubic domains for both the prostate models and the 3D masks were generated using the open-source visualisation software Paraview (more details are provided in the Supplementary Material).

**TABLE 1:** Data of the Dice similarity index, D , calculations to quantify the agreement between the simulated prostate model, *in silico*, and the segmented clinical imaging data, TRUS-END.

*Simulation settings*	PAT1	PAT2
Without effect of anchor needles	0.854	0.863
Accounting for the effect of anchor needles	0.931	0.919

Simulating the needle insertion process led to non-negligible deformation of the prostate geometry for the two patients. For PAT1, mean in-plane displacement was evaluated at 1.2±0.8 *mm* while maximum in-plane displacement was 4.3 *mm*. For PAT2, mean in-plane displacement was 1.2±0.7 *mm* while maximum in-plane displacement was 3.7 *mm*.

### 3.2 Importance of modelling anchor needles

**FIGURE 6:**
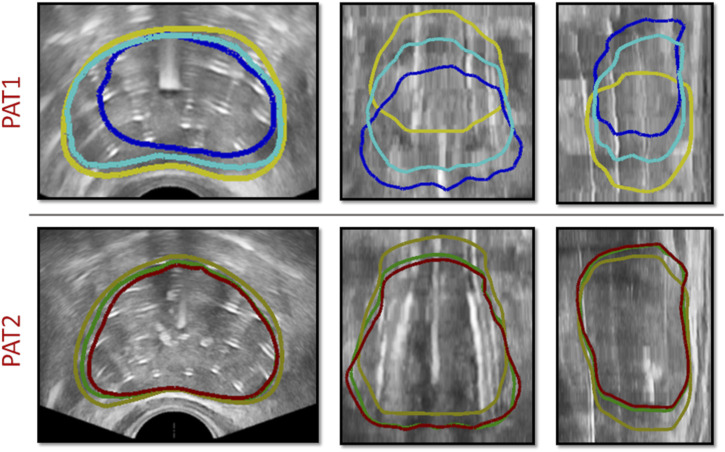
(*top row*) HDR-BRT simulations for PAT2 overlaid on top of the TRUS-END images to investigate the biomechanical effect of anchor needles in the prostate deformation predictions. Yellow solid curves outline the undeformed prostate, while the blue contours show the simulated deformed prostate without accounting for the anchor needles biomechanical effect, and cyan contours show the simulated deformed prostate for when the anchor needles effect was accounted in the simulation. (*bottom row*) HDR-BRT simulations for PAT1 using two different sequences for needle insertion. As above, yellow solid curves outline the undeformed prostate (i.e., from TRUS-0), while the coloured contours show the simulated deformation for the two different configurations: green contours correspond to the first placement sequence and red contours correspond to the second placement sequence of catheter-needles.

To assess the importance of accounting for anchor needles in the simulations, *in silico* HDR-BRT tests were run with or without modelling their biomechanical influence on the prostate deformation. Figure 6 (top row) depicts the effect of anchor needles installation in the prostate shape for PAT2, while Table 1 lists the Dice similarity index that was calculated for the two modelling scenarios for both patients.

### 3.3 Influence of needles’ sequence of insertion

Another interesting aspect interrogated, was the sequence by which catheter-needles were inserted in the prostate and whether this impacted the simulation results and the final outcome with regards to the deformation of the organ. As such, a simulation of the HDR-BRT procedure was rerun with a different sequence for the needles inserted into the prostate tissue. The original positioning began with a catheter on the left bottom side of the ultrasound probe (in the short-axis view), continued with a catheter on the right top side of the ultrasound probe and continued in an alternate manner towards the centre. The second catheter-needles’ installation sequence followed a similar alternate pattern but the catheters were inserted from bottom right and continued to bottom left, etc. Figure 6 (bottom row) demonstrates the difference between the simulated deformation for when a different sequence was assumed for PAT1. Additionally, a quantitative comparison between the two brachytherapy simulation scenarios resulted to the following Dice similarity index results: $D_{1}=0.925$ and $D_{2}=0.931$ , respectively.

## 4 Discussion

This work has focused on developing a three-dimensional *in silico* framework to model prostate biomechanics, and utilising the framework to produce physiologically realistic simulations of HDR-BRT on a patient-specific basis. Despite numerous published works which have successfully modelled the insertion of a single needle, the challenging process of simulating the entire HDR-BRT procedure that involves the insertion of multiple catheters, has not been investigated yet. To the best of our knowledge, this is the first work to simulate the insertion of multiple catheters in soft biological tissues, and importantly, a large number of them (2 anchor needles, 17 catheter-needles). Additionally, this work has paid particular attention to producing personalised simulations of brachytherapy, by incorporating patient-specific organ geometries and boundary/loading conditions. Notably, the study relied on comprehensive medical images acquired during HDR-BRT, which have provided a unique opportunity for model evaluation–an important prerequisite for clinical translation of medical simulators.

Utilising such data enabled the evaluation of the proposed *in silico* framework and the assessment of the validity of the modelling assumptions. For instance, qualitative and quantitative comparisons (Figure 6; Table 1) highlighted the importance of accounting for the constraining effect of the two anchor needles. Comparisons of the *in silico* results against prostate image segmentation masks from TRUS-AN suggest that our approach for simulating the effect of anchor needles produces accurate prostate deformation predictions (first and third row in Figure 5). Although the available data (before HDR-BRT, after the insertion of the anchor needles and following the insertion of all catheters) did not allow for a mechanistic understanding of the constraining effect of anchor needles, a follow up study could be directed towards acquiring TRUS snapshots after each needle insertion–this could enable a mechanistic model to describe the anchor needles’ influence. Similarly, the proposed *in silico* framework was used to probe the sensitivity of the model in the sequence by which the catheter-needles were inserted in the prostate. As the exact sequence followed in the HDR-BRT treatments was not available, two different placement sequences were tested (Figure 6). The difference between the two scenarios was marginal; however, further studies are warranted that could identify optimal catheter insertion sequences. Furthermore, the presented modelling framework predicted non-negligible prostate deformation (maximum in-plane displacements in the order of 4 *mm*), highlighting the need for accounting for needle-induced deformations during HDR-BRT. Future work could consider the acquisition of complementary data for a small cohort of patients (e.g., tagged MRI, target contouring at different stages of HDR-BRT) to offer a quantitative assessment of the accuracy in target localisation in HDR-BRT and how that can be improved by the proposed *in silico* approach.

With regards to the limitations of the proposed *in silico* framework, the prostate reference configuration (also known as “stress-free” or “zero-pressure” configuration) was unknown, therefore, the image frame TRUS-0 was utilised for the computational unloaded domain. However, the prostate depicted in TRUS-0 was deformed by the ultrasound probe, while surrounding organs were also likely to exert stresses on the tissue. As the choice of reference configuration is a known issue in soft tissue biomechanical modelling (Vavourakis et al., 2016; Hadjicharalambous et al., 2021), it is likely that slightly different deformation outcomes would have been predicted with adopting a another reference configuration, e.g., the MRI setting. Additionally, the Neo-hookean material law was employed within, and the material parameters were set to specific values. Prospective work will consider testing different constitutive laws, e.g., Mooney-Rivlin, Ogden, etc., as the Neo-hookean law has exhibited limitations in large-strain problems, while also being unsuitable for anisotropic tissues. Nevertheless, the material law and specific material parameters used are not expected to substantially alter the results (Wittek et al., 2020) due to the specific formulation employed. Moreover, while the grid size was selected based on earlier convergence studies Wittek et al. (2020), a detailed convergence analysis would be a valuable future direction to ensure that model results are independent of the discretisation level.

Furthermore, while image resolution is satisfactory throughout all stages of HDR-BRT, as the needle insertion process progresses in HDR-BRT, the quality of TRUS image gradually deteriorates, rendering the processing step to accurately segment the prostate geometry very challenging. While a clear delineation of the prostate region is straightforward from TRUS-0 – the organ delineation facilitates the generation of physiologically accurate computational grids (3D of model) of the prostate–inevitably, this deterioration in image quality imposes an uncertainty on the assessment of model accuracy towards the end of the HDR-BRT simulations. To overcome this issue, prospective work could be directed towards acquiring post-HDR-BRT MRI scans, and notably towards performing the entire HDR-BRT procedure within an MRI scanner using magnetic field-compatible needles. The superior quality of the MRI will aid towards improving model accuracy and further support the *in silico* framework validation. Future work can also consider all image segmentation being performed by multiple experienced clinicians in order to examine intra- or inter-observer variability and assess its influence on the accuracy of the HDR-BRT simulation results. Additionally, taking advantage of the rich datasets available, multi-modal image fusion could be considered for facilitating image segmentation.

Finally, only two patient cases were modelled, yet plans are already in place for extending the study to a much larger cohort of patients. The extension of this proof-of-concept study will be invaluable for examining the predictive capacity of the presented approach and assessing its reproducibility potential. Notably, a much larger cohort will be needed for a rigorous validation of the proposed work, while multi-centre studies or comparisons to *in vitro* experiments and clinical trials could be considered in the future. Additionally, a larger number of cases will allow for a comprehensive sensitivity analysis, to identify the impactful variables and thus improve model accuracy and generalisation capacity. Nevertheless, the low number of cases studied in this preliminary enabled the systematic exploration and development of the modelling framework used and provided insightful insights (e.g., on the effect of anchor needles, the relative stiffness of surrounding domain, the sequence of needle insertion) which will be valuable for future studies in the field.

The proposed modelling and data-processing procedure demonstrated its capacity to produce physiologically realistic predictions of the prostate deformation during HDR-BRT (see Figure 5; Table 1). We envision that such a modelling could be particularly useful in the clinical practice, whereby the current pre-operative planning assumes no needle-induced deformations to the prostate and the surrounding tissue. Contrary to this, our proposed modelling approach could allow the treating physician to visualise the anatomical changes predicted by the *in silico*framework and make an informed decision on possible adjustments to the “pre-plan.” Visualisation of the expected deformation will therefore enhance the precision of needle placement during HDR-BRT while minimising the manual correction required ‘on the fly’ during the procedure. The modelling framework could also form the basis for inverse modelling, whereby the optimal needle position could be calculated, taking into account the possible induced deformation. Thus, the proposed *in silico* framework holds great potential for clinical translation as a pre-operative planning tool, to assist in designing the HDR-BRT procedure and optimising the delivery of the radioactive material. For a seamless integration of the *in silico*model into the existing workflow of HDR-BRT, the model would ideally need to be integrated into the clinical pre- and intra-operative planning system (e.g., GOC uses Elekta’s software platform *Oncentra Prostate*). Alternatively, a framework should be set up to allow the *in silico*model to communicate and exchange data with the clinical planning system. Moreover, the framework could be readily integrated with haptic robotic technology for use as a training tool for inexperienced practitioners, i.e., to comprehend the effect of catheter-induced deformation in prostate brachytherapy.

## 5 Conclusion

This paper presented an *in silico* modelling platform for patient-specific simulations of needle insertion during HDR-BRT for prostate cancer. An important novelty of this contribution was attempting physiologically-relevant simulations of HDR-BRT involving the insertion of multiple needles–a challenging, yet necessary, part of the process which has not been explored in the literature. Additionally, this study utilised comprehensive medical imaging data acquired prior, during and after HDR-BRT–such data are invaluable for informing and personalising the *in silico* model, as well as for providing directions for improvement during model evaluation. Importantly, such data are vital for model validation–a necessary step towards clinical translation. The proposed *in silico* framework for personalised HDR-BRT simulations holds substantial potential to be enhanced into a training tool or a pre-operative simulator to boost the accuracy and efficiency of HDR-BRT.

## Data Availability

The original contributions presented in the study are included in the article/Supplementary Material, further inquiries can be directed to the corresponding author.
