# Supplementary material for: Personalised in silico biomechanical modelling towards the optimisation of high dose-rate brachytherapy planning and treatment against prostate cancer
Source: Front Physiol. 2024 Oct 24;15:1491144. doi: 10.3389/fphys.2024.1491144 (PMC11540655; doi:10.3389/fphys.2024.1491144)
Supplement: Supplementary file 1 [file DataSheet1.pdf]

# Supplementary Material

## Personalised *in silico* biomechanical modelling towards the optimisation of high dose-rate brachytherapy planning and treatment against prostate cancer

M. Hadjicharalambous, Y. Roussakis, G. Bourantas, E. Ioannou, K. Miller, P. Doolan, I. Strouthos, C. Zamboglou, V. Vavourakis

## Overview of the HDR-BRT procedure

High dose-rate brachytherapy (HDR-BRT) involves the transperineal implantation of several catheter needles into the patient's prostate, which facilitate the temporary placement of a radioactive source inside the tumour site. Catheter placement was conducted under real-time image guidance, commonly through transrectal ultrasound (TRUS), using a continuous probe movement technique, allowing catheter positions to be manually adjusted. Pre-operative planning included contouring (also referred as TRUS segmentation) of the patient's prostate, the urethra and anterior rectal wall. This task was performed by a specialist physician (radiation oncologist, urologist, or radiologist), using as a guide a higher-resolution magnetic resonance imaging (MRI) data acquired on the same day. A pre-operative plan was then performed by a radiology clinician using the TRUS segmentation, in which the planner selects the positions for catheter insertion to enable optimal distribution of radiation dose to the prostate, with minimal damage to surrounding tissues. The procedure began with the insertion of two anchor needles – these are thinner and shorter than the catheters delivering the radioactive fluid – that were inserted and anchored on the prostate to stabilise it during the forthcoming catheter insertion procedure. Subsequently, the insertion of the catheter-needles used for radiation commenced, involving the sequential insertion of multiple catheters (commonly 16—20 catheters in total). The catheters used in HDR-BRT are metallic and relatively stiff, to minimise deflection. This part of the procedure could often include manual repositioning (retraction and re-insertion) of catheters to correct deviations of catheter placement from the pre-operative treatment plan. Once

all catheters were inserted, a dosimetric plan was prepared to determine the optimal dwell times and active source positions for the radioactive source, using the Hybrid Inverse Planning and Optimisation algorithm, e.g., as in (Karabis et al., 2006). Dose specification was given as the mean dose on the planning target volume, defined as the entire prostate gland without margins (Tselis et al., 2013). The radiation was then delivered to the tumour site using a computer-controlled system and, finally, the catheters were removed.

## Medical imaging data acquisition

Medical images were acquired during HDR-BRT for two prostate cancer patients (PAT1 and PAT2) that were randomly selected from a male patient cohort with intermediate risk for neoplasia in the prostate. Both patients were admitted at the German Oncology Centre (GOC) in Limassol, Cyprus, and received HDR-BRT as part of their clinically selected therapeutic strategy. Patients also signed a written consent form allowing use of their fully anonymised data (including medical images) to be used for research and development purposes, in the interest of public health. This was in full compliance with GDPR regulations, in accordance to the EU's General Data Protection Regulation Article 6(4). Use of the medical imaging data for this study was approved by the National Bioethics Committee in Cyprus (Application ID: EEBK EΠ2020.01.246).

Medical images used in this study included (*i*) an MRI scan that was acquired prior to brachytherapy, (*ii*) a TRUS scan that was acquired during brachytherapy and right before initiating transperineal anchor needle and catheter-needle insertion (labelled hereafter as TRUS-0), (*iii*) a TRUS scan that was acquired right after the two anchor needles were positioned (labelled hereafter as TRUS-AN) and, finally, (*iv*) a TRUS scan that was acquired at the end of the procedure with all catheters inserted (labelled hereafter as TRUS-END). The MRI data used were T2-weighted Fast Spin Echo sequences of the pelvic region around the prostate, with a resolution of $1024 \times 1024 \times 48$ pixels, a 0.2148 $mm$ in-plane resolution and a 1.5 $mm$ slice thickness – all pertinent data were acquired on a GE Signa 1.5 Tesla MRI scanner. All TRUS imaging data acquisitions were carried out using a BK Medical bk3000, with an endocavity biplane transducer.

## Model personalisation

Initially, spatial registration of the medical images, i.e., magnetic resonance imaging (MRI) and transrectal ultrasound (TRUS) scans, was performed to enable consistent use of all imaging data (i.e., to align the prostate domain from different image modalities). A subsequent step was the segmentation of the prostate geometry from the anatomical TRUS images, which involves

the contouring of the prostate region. The segmentation of TRUS-0 was essential since the segmented prostate domain was used to build the 3D prostate 'digital' model. Additionally, segmentation of the TRUS-AN and the TRUS-END imaging data was performed – these were used for *in silico* model evaluation purposes.

## Prostate medical imaging registration

Spatial image registration was performed to enable the reliable and consistent use of all available medical imaging modalities for the *in silico* modelling work alignment of the prostate domain in all images. Specifically, MRI scans, (TRUS-AN) and (TRUS-END) imaging data were registered to the corresponding TRUS-0 imaging data acquired before any needle placement. The rigid registration algorithm available in the image computing platform 3D Slicer[1] (Fedorov et al., 2012) was employed. A representative example of rigid registration is illustrated in Figure 1 for PAT1, where the TRUS-0 image is overlaid on top of a slice of the MRI sequence.

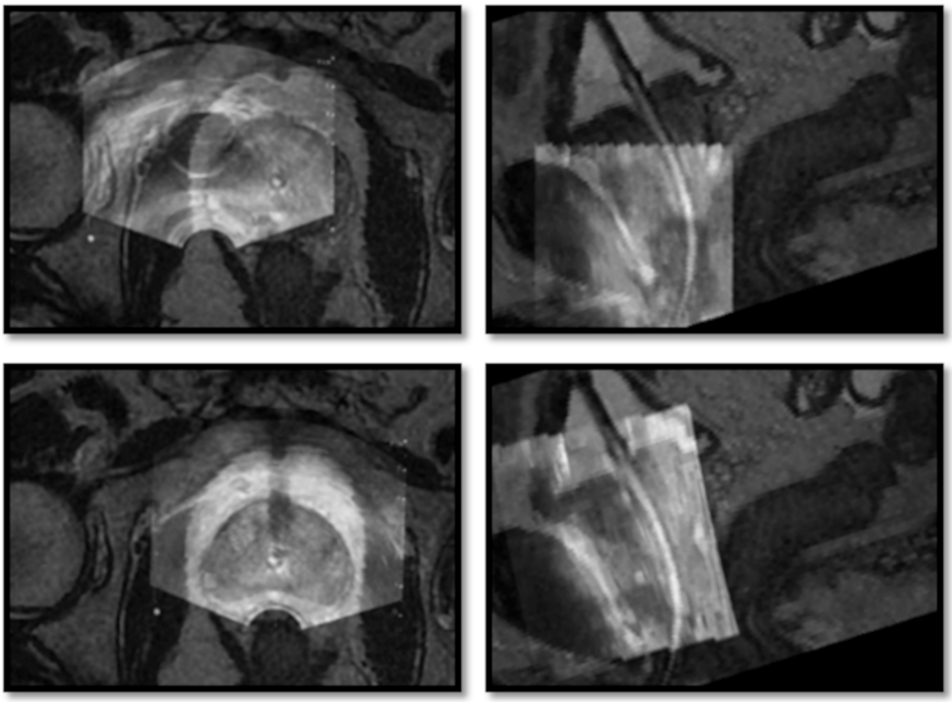

Figure 1: Two different views of the TRUS-0 image overlaid on top of the MRI, both images acquired prior to needle insertion, before (*top row*) and after (*bottom row*) rigid registration was performed for the PAT1 image data.

[1] `https://www.slicer.org`

## Prostate medical imaging segmentation

Segmentation of the prostate geometry from TRUS-0 was performed as part of the clinical HDR-BRT procedure by a radiation oncology specialist at GOC using the clinical treatment planning system (Oncentra Prostate, Elekta AB) available at the clinical centre. More specifically, the clinical team at GOC utilised the software Oncentra Brachy[2] for the prostate data contouring, the dose volume histogram planning and the region-of-interest management for the brachytherapy procedure.

The MRI data were used as guidance for the delineation of the prostate and the urethra on the TRUS-0 data – typically MRIs illustrate better contrast amongst the different tissue types than that of TRUS images. The clinical segmentations of TRUS-0 were then smoothed and refined, as shown in Figure 2 of the manuscript, to enable their use for the *in silico* modelling work using the specialised image processing software ITKSnap[3]. The software was also used for the manual segmentation of the TRUS-AN and TRUS-END images.

Additionally, the location of the catheters was identified by manually delineating the catheters inserted into the prostate tissue, in TRUS-AN and TRUS-END images. This was key in the model personalisation step, as the locations for catheter insertion served as 'loading conditions' for the HDR-BRT simulations. Catheter segmentation in TRUS-END for PAT2 is shown in Figure 2 of the manuscript. The depth each catheter had penetrated within the prostate geometry was also determined from the catheters' segmentation of TRUS images.

## Personalised prostate 3D model generation

The computational domains used to carry out the simulations for each patient case (PAT1 and PAT2) were generated using the prostate segmentations from the respective TRUS-0 images. For each case, three-dimensional unstructured meshes of four-node tetrahedral elements were generated that capture the prostate gland and the surrounding tissue. The grids were produced using the open-source meshing software MeshLab[4] and Gmsh[5] for the geometry manipulation and refinement and for the three-dimensional mesh / point-cloud generation, respectively. For each prostate model case, a cubic domain was created around the prostate geometry that defined the control volume of the *in silico* model, in order to confine the tissue domain of analysis, as shown in Figure 2. Details on the grids are shown in Table 1, with grid size selected based on previous convergence analysis (Wittek et al., 2020).

[2])https://www.elekta.com/products/brachytherapy/oncentra-brachy/ (v4.2.3.11)
[3]http://www.itksnap.org/pmwiki/pmwiki.php
[4]https://www.meshlab.net
[5]https://gmsh.info

The cubic 'external boundary' was used to impose the boundary conditions, as explained below, and was positioned adequately distant from the prostate geometry such that the boundary will not impact the simulation results (of tissue displacements and deformation) on the region of interest, i.e., the prostate gland. Thus, the edge of the cubic domain was set approximately two times larger than the corresponding bounding box of the prostate geometry.

| *Personalised computational grids* | PAT1 | PAT2 |
|---|---|---|
| Number of nodes | 15,049 | 14,030 |
| Number of integration points | 79,006 | 73,491 |

Table 1: Nodes and integration points in the personalised computational grids (3D models) for PAT1 and PAT2

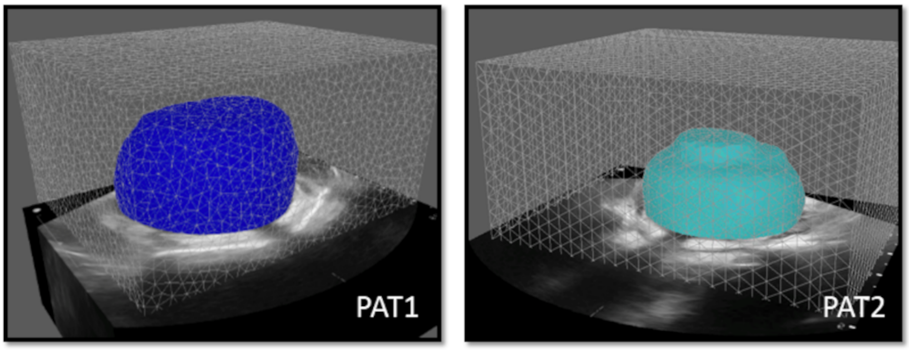


Figure 2: Personalised computational grids (3D models) for PAT1 (*left*) and PAT2 (*right*) respectively, overlaid on top of the respective TRUS-0 images.

## Boundary conditions

Dirichlet boundary conditions were applied on the cubic domain (the control volume) of the prostate and the surrounding tissue – the external outline of the analysed domain and the geometry of the prostate gland is shown in Figure 3. In particular, the free boundary opposite to needle insertion direction was constrained from moving in the corresponding normal direction (i.e., parallel to the needle insertion direction; green side of the analysed domain in Figure 3), while in-plane displacements were set to zero on two opposite sides of the cube being parallel to the direction of needle insertion (blue sides of the domain in Figure 3). Anchor needle and catheter insertion was simulated as described in the above section that describes the modelling approach for the insertion of an individual needle.

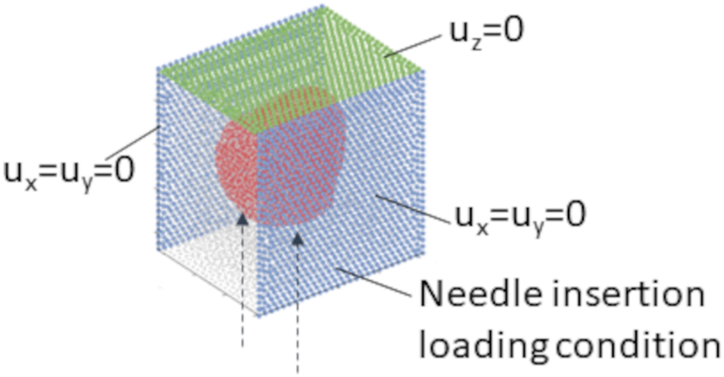


Figure 3: Schematic of the boundary conditions applied, with needle insertion assumed to occur in the vertical direction (here the z-axis).

### Material model parameters

In light of McAnearney et al. (2011), the employed kinematic approach is numerically non-sensitive to the model material parameters, which were observed to not affect the predicted displacements (Wittek et al., 2020, 2008). A higher stiffness value was used for the cubic region surrounding the prostate (10 times stiffer), to account for potential stiffer parts in this region (e.g., bones). At the same time, a much higher stiffness value ($\sim$ 30 times stiffer) was used for simulating the effect of anchor needles and the effect of catheters already placed in the prostate geometry.

## Modelling prostate biomechanics and needle insertion

The mechanical deformation of the prostate tissue following catheter insertion was formulated as a quasi-static, nonlinear continuum mechanics problem governed by the linear momentum equation: $\boldsymbol{\nabla} \cdot \boldsymbol{\sigma} = 0$, with $\boldsymbol{\nabla}$ and $\boldsymbol{\sigma}$ being the gradient operator and Cauchy stresses respectively, where both inertial and body forces were considered negligible. The partial differential equation was discretised and solved numerically using an element-free Galerkin methodology (Belytschko et al., 1994), as this framework has proven numerically superior for needle insertion simulations. Due to the very large deformations and geometric discontinuities observed during subcutaneous needle insertion in soft tissue, conventional finite element methods often struggle to converge to a solution, whereas the element-free Galerkin method has proven versatile for simulations involving large strains and moving boundaries (Horton et al., 2010). Importantly, mesh-free (element-free, or meshless) methodologies alleviate the strict requirements for mesh quality and remeshing often accompanying the finite element method, and allow

for faster generation of 3D personalised models and computational grids – a necessary feature for a simulation tool of potential clinical use. Thus, a mesh-free numerical method was employed in this study that builds on the meshless total Lagrangian explicit dynamics method of Bourantas et al. (2021).

## Insertion of a single needle modelling approach

Needle insertion (i.e., referring to the penetration of either anchor needles or catheter-needles) was modelled using a kinematic approach that builds upon the model proposed by Wittek et al. (2020). Wittek and colleagues proposed an empirical relation of catheter puncture and penetration, validated against experimental data using phantoms. In the original formulation, needle insertion was simulated by defining a region of needle influence, whereby all nodes (of the solid body) within a support region displace following the movement of the tip of the needle. In particular, all points within a cylindrical domain of influence of the needle ('IN', referred to here as influenced nodes) were set to displace by a fraction of the incremental penetration depth of the needle tip ('NT'). Thus, the displacement of a node within the region of influence at time-step $t$, $u_t^{\mathrm{IN}}$, in the direction of needle insertion, can be expressed with respect to the displacement of the needle tip at time-step $t$, $u_t^{\mathrm{NT}}$, and a scaling factor parameter, $C_D$, that is empirically derived from phantom experiments (Wittek et al., 2020), and reads:

$$u_t^{\mathrm{IN}} = u_{t-1}\, C_D\left(u_t^{\mathrm{NT}} - u_{t-1}^{\mathrm{NT}}\right), \tag{1}$$

where both $u_t^{\mathrm{IN}}$ and $u_t^{\mathrm{NT}}$ refer to the displacements in the direction of the needle insertion path.

Preliminary numerical experiments (not presented herein) employing the original formulation of the kinematic approach (Eq. 1) led to substantial deformation during needle insertion which was not reflected in the TRUS images acquired after needle insertion. This discrepancy motivated two important modifications in the kinematic condition. Firstly, rather than assuming that all nodes that fall within the cylindrical domain of influence of the needle are affected uniformly by the motion of the tip of the needle, a depth-dependence relation was introduced. Particularly, the displacement of a node that falls within the cylinder of needle influence was assumed to depend on the node's relative distance from the needle tip. Additionally, as a linear dependence depth-dependence was shown to induce more pronounced deformation compared to the data-derived prostate geometry, a polynomial relationship was introduced to account for the depth-dependence:

$$u_t^{\mathrm{IN}} = u_{t-1}^{\mathrm{IN}}\, C_D\left(u_t^{\mathrm{NT}} - u_{t-1}^{\mathrm{NT}}\right)\left(1 - \sqrt{|x_t^{\mathrm{NT}} - x_t^{\mathrm{IN}}|/\sqrt{d_n}}\right), \tag{2}$$

where $|x_t^{\mathrm{NT}} - x_t^{\mathrm{IN}}|$ refers to the distance in the direction of needle insertion between a node in the region of influence and the needle tip. This expression permits points closer to the needle/catheter tip to have a more pronounced displacement compared to the corresponding points that are further away from the tip of the penetrating body. Parameter $d_N$ introduces the degree of depth dependence and was set as a fraction of the maximum depth of needle penetration. The maximum depth is a patient-specific parameter derived from the medical images for each patient through manual segmentation.

One final important modification, was to further restrict the region affected by the needle. The parameter $d_N$ served as a threshold, whereby only nodes within a distance lower than the threshold to the needle tip are assumed to be affected by needle tip motion. In this case, all affected nodes are again displaced based on a polynomial distance dependence in Eq. 2.

## Dice similarity index for simulation output data quantification

The spatial comparison between the simulated 3D prostate model and the segmented clinical imaging data is performed through the Dice similarity index, $D$, which is used to measure the spatial overlap agreement of the two volumes. As explained in the manuscript, the prostate organ was delineated manually from the ultrasound imaging scans at the end of anchor and catheter needles insertion (TRUS-AN and TRUS-END). For model evaluation purposes, these segmentations were considered as the ground truth. In general, the Dice index measures the similarity between two sets by calculating the overlap between the predicted and actual volumes, is a strict measure of accuracy and provides a score ranging from 0 (no overlap) to 1 (perfect overlap). Using the definition of true positive (TP), false positive (FP), true negative (TN) and false negative (FN), the Dice similarity index is defined by

$$D = \frac{2 \times (\mathtt{TP})}{2 \times (\mathtt{TP}) + (\mathtt{FP}) + (\mathtt{FN})}\,, \tag{3}$$

while a graphic illustration of $D$ is provided in Figure 4 below. This index focuses on the precise overlap of the two volumes (*in silico*-predicted and segmented) and it neglects situations where the two volumes are in the same general area but have little overlap; thus, the Dice similarity index is considered a strict metric to assess image similarity.

In order to calculate the Dice index, $D$, and carry out the comparison between the 3D prostate model simulation results and the segmented prostate from the TRUS-END scan, the simulated domain was transformed into an image of similar size to the data using the open-source software Paraview,[6]

[6]`https://www.paraview.org`

and a common cubic image domain was adopted for both cases. More specifically, a bounding box was constructed for each dataset separately, to fully encase the individual volume of interest. Subsequently, the largest in size bounding box was adopted for the computation of the Dice similarity index, $D$.

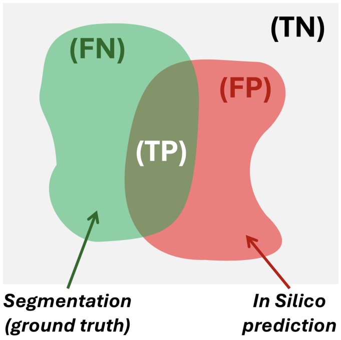


Figure 4: Schematic of the region of interest, the delineated tissue compartment/organ from the image segmentation (ground truth) and the *in silico*-predicted deformed tissue compartment/organ.

# References

T. Belytschko, Y. Y. Lu, and L. Gu. Element-free Galerkin methods. *International Journal for Numerical Methods in Engineering*, 37(2):229–256, 1994. doi: 10.1002/nme.1620370205. URL `https://doi.org/10.1002/nme.1620370205`.

G. Bourantas, B.F. Zwick, G.R. Joldes, A. Wittek, and K. Miller. Simple and robust element-free Galerkin method with almost interpolating shape functions for finite deformation elasticity. *Applied Mathematical Modelling*, 96:284–303, 2021. doi: 10.1016/j.apm.2021.03.007. URL `https://doi.org/10.1016/j.apm.2021.03.007`.

Andriy Fedorov, Reinhard Beichel, Jayashree Kalpathy-cramer, Julien Finet, Jean-christophe Fillion-robin, Sonia Pujol, Christian Bauer, Dominique Jennings, Fiona Fennessy, Milan Sonka, John Buatti, Stephen Aylward, James V Miller, Steve Pieper, and Ron Kikinis. 3D Slicer as an image computing platform for the Quantitative Imaging Network. *Magnetic Resonance Imaging*, 30(9):1323–1341, 2012. ISSN 0730-725X. doi: 10.1016/j.mri.2012.05.001. URL `http://dx.doi.org/10.1016/j.mri.2012.05.001`.

Ashley Horton, Adam Wittek, Grand Roman Joldes, and Karol Miller. A meshless total lagrangian explicit dynamics algorithm for surgical

simulation. *International Journal for Numerical Methods in Biomedical Engineering*, 26:977–998, 8 2010. doi: 10.1002/cnm.1374. URL https://doi.org/10.1002/cnm.1374.

Andreas Karabis, Stavroula D Giannouli, and Dimos Baltas. 40 HIPO: A hybrid inverse treatment planning optimization algorithm in HDR brachytherapy. *Radiotherapy and Oncology*, 76(2):S29, 2006. doi: 10.1016/S0167-8140(05)81018-7. URL http://doi.org/10.1016/S0167-8140(05)81018-7.

Stephen McAnearney, Andriy Fedorov, Grand R. Joldes, Nobuhiko Hata, Clare Tempany, Karol Miller, and Adam Wittek. The Effects of Young's Modulus on Predicting Prostate Deformation for MRI-Guided Interventions. In Adam Wittek, Poul M.F. Nielsen, and Karol Miller, editors, *Computational Biomechanics for Medicine*, pages 39–49, New York, NY, 2011. Springer New York. ISBN 978-1-4419-9619-0. doi: 10.1007/978-1-4419-9619-0_5. URL https://doi.org/10.1007/978-1-4419-9619-0_5.

Nikolaos Tselis, Ulf W Tunn, Georgios Chatzikonstantinou, Natasa Milickovic, Dimos Baltas, Markus Ratka, Zamboglou Whitcomb, and Nikolaos. High dose rate brachytherapy as monotherapy for localised prostate cancer: a hypofractionated two-implant approach in 351 consecutive patients. *Radiation Oncology*, 8:1–7, 2013. doi: 10.1186/1748-717X-8-115. URL http://doi.org/10.1186/1748-717X-8-115.

A. Wittek, T. Dutta-Roy, Z. Taylor, A. Horton, T. Washio, K. Chinzei, and K. Miller. Subject-specific non-linear biomechanical model of needle insertion into brain. *Computer Methods in Biomechanics and Biomedical Engineering*, 11(2):135–146, 2008. ISSN 10255842. doi: 10.1080/10255840701688095. URL http://doi.org/10.1080/10255840701688095.

Adam Wittek, George Bourantas, Benjamin F. Zwick, Grand Joldes, Lionel Esteban, and Karol Miller. *Mathematical modeling and computer simulation of needle insertion into Soft Tissue.* 2020. doi: 10.1371/journal.pone.0242704. URL http://dx.doi.org/10.1371/journal.pone.0242704.
